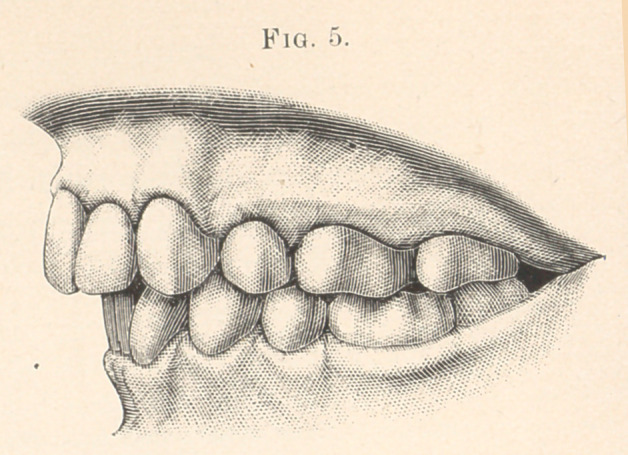# Twisted Wire for Regulating Teeth

**Published:** 1893-04

**Authors:** William Slocum Davenport

**Affiliations:** Paris, France


### TWISTED WIRE FOR REGULATING TEETH.

BY WILLIAM SLOCUM DAVENPORT, D.D.S., PARIS, FRANCE.

Fig. 1 represents the mouth of a young lady, seventeen to eighteen years of age.

The superior teeth articulate with those of the lower jaw,—one cusp is too far forward, and the inferior front teeth are flattened backward, while their cutting-edges arch upward until the incisors nearly touch the palate.

The bicuspid region in the lower arch is broad enough to conform to, and permit the teeth to articulate with, those of the upper jaw.

The vault is very high.

The upper lip covers about one-fourth the upper teeth, and the lower lip falls far back and under the superior incisors.

In the history of the case we find,—

1. The patient, until four years old, had the habit of sucking her thumb, with its palm side placed against the roof of the mouth.

2. She was a constant sufferer from adenoid growths and bronchitis, on account of which the tonsils had been incised at the twelfth year.

3. She was a mouth-breather.

4. No similar deformity could be found among any of her relatives.

The first means employed towards correcting the irregularity was to push forward the lower incisors by the use of *linen tapes*, acting as wedges between these teeth and a corresponding edge of a plate which was fitted over the molars and bicuspids.

When this was accomplished a simple rubber retaining-plate was inserted, and the patient left Paris for the winter.

It was my intention, upon the patient's return, to spread the upper arch and attempt to jump the bite, but it was finally deemed more practicable in the *present* case to draw the upper teeth backward, and, to obtain the necessary space, the two superior first bicuspids were extracted. An appliance (Fig. 2) was then made, consisting of a rubber plate, which covered the upper back teeth. Into the right side of the plate was vulcanized one end of a half-round platinum wire, which was passed around in front of the incisors and terminated in a loop at the free end. Two little hooks were soldered to the front of the band in such a way as to catch over the ends of the centrals when the plate was in the mouth, and prevent the wire slipping up against the gums. Into the left side of the plate a staple was vulcanized.

When the plate was in position a copper wire was passed through both the loop and staple, and had its ends brought together and twisted, this producing pressure upon the centrals, laterals, and cuspids.

From time to time another twist was given to the copper wire, until, at the end of seven weeks, the teeth were in the desired position.

A retaining fixture was then placed, consisting of a strip of pure gold, No. 5 to 6 Stubbs, and French gauge, so bent and soldered as to form a loop at each end.

Having previously separated the teeth with linen tapes, the looped strips were covered inside with thick chlora-percha and passed around the anchor-teeth, allowing the loops to be on the outer sides.

Copper wire was passed through these loops, and the ends of the wires were brought together and twisted (Fig. 3) until the pure gold bands were perfectly swedged to the convexity of the crowns, forcing the superfluous chlora-percha out at all points and making an accurate fit. (These bands did not move until taken off four months later.)

Copper wires were fastened to the loops left in the band at the

Fig. 1.

Fig. 5.

Fig. 2.

Fig. 3.

Fig. 4.

buccal surfaces of the teeth, and brought around the front teeth from both sides and twisted together at the centrals. This drew the six front teeth to their exact places. The twisted ends were then bent over the cutting-edges of the centrals, to protect the gums from the wire. All rough places at the sides were then covered with gutta-percha.

Fig. 4 shows retainer in position.

By comparing Fig. 5 with Fig. 1, we find the six front teeth were drawn directly backward.

The patient was instructed to remove the plate while eating, and by so doing a very good articulation at the finish was secured.

Positive and intermittent force is secured by such a use of twisted wires as above indicated. The principle involved is that of the inclined plane, which is also the principle made use of in the screw.

Appliances dependent upon twisted wire for the application of force are easily made and applied, and possess many obvious advantages over the screw in very many cases. Wire of silver, galvanized iron, or copper, owing to their pliability, strength, and cheapness, will be found very satisfactory.

## Figures and Tables

**Fig. 1. f1:**
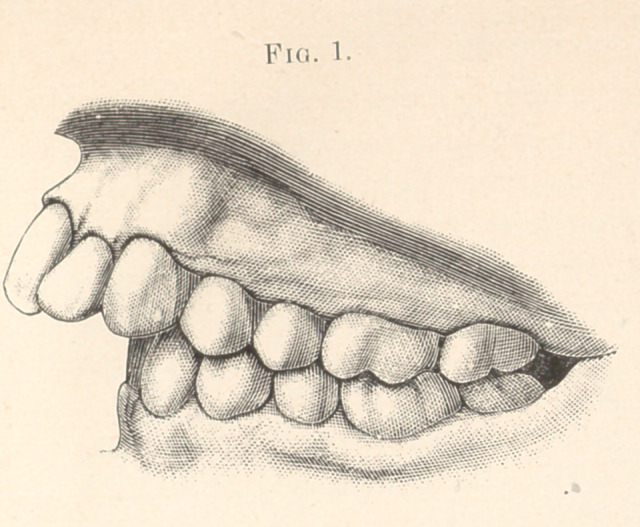


**Fig. 2. f2:**
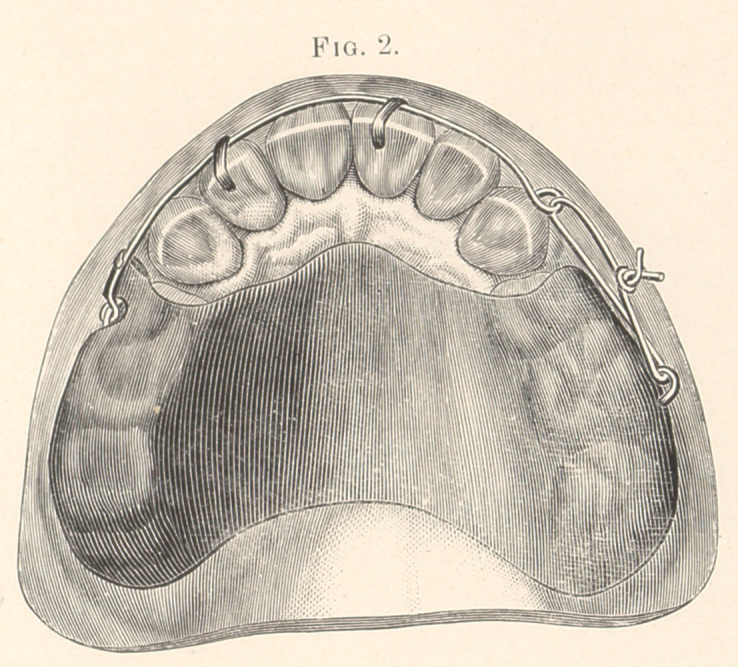


**Fig. 3. f3:**
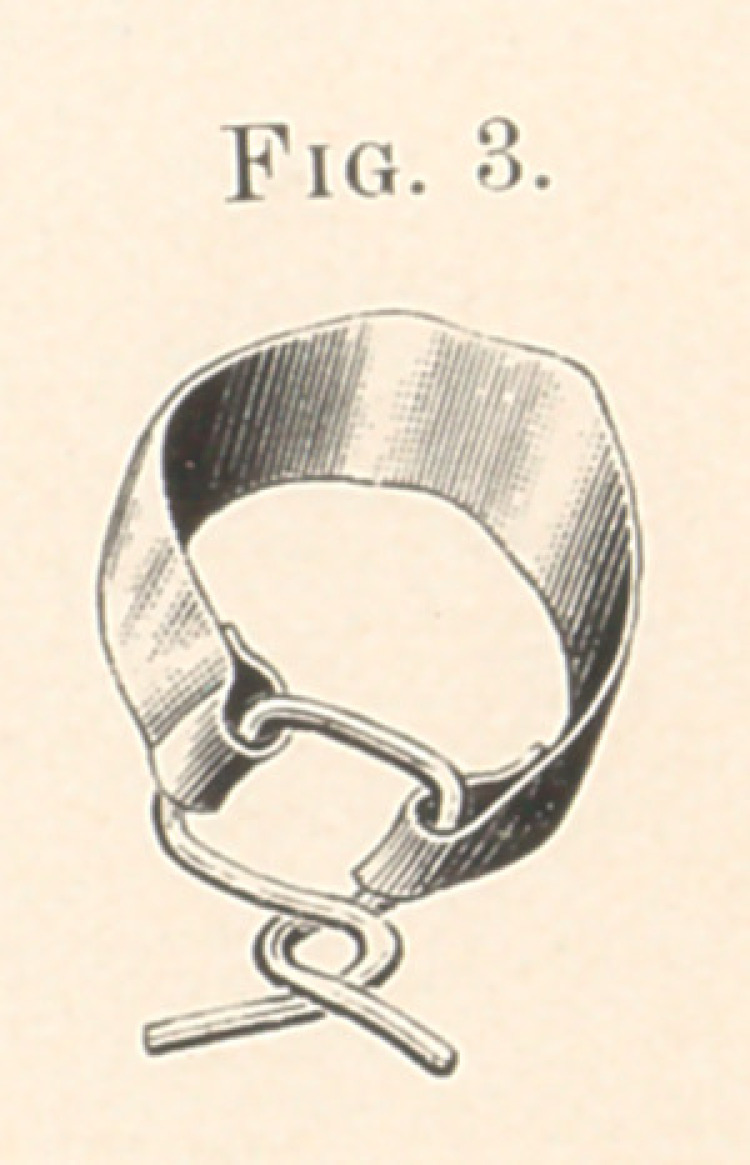


**Fig. 4. f4:**
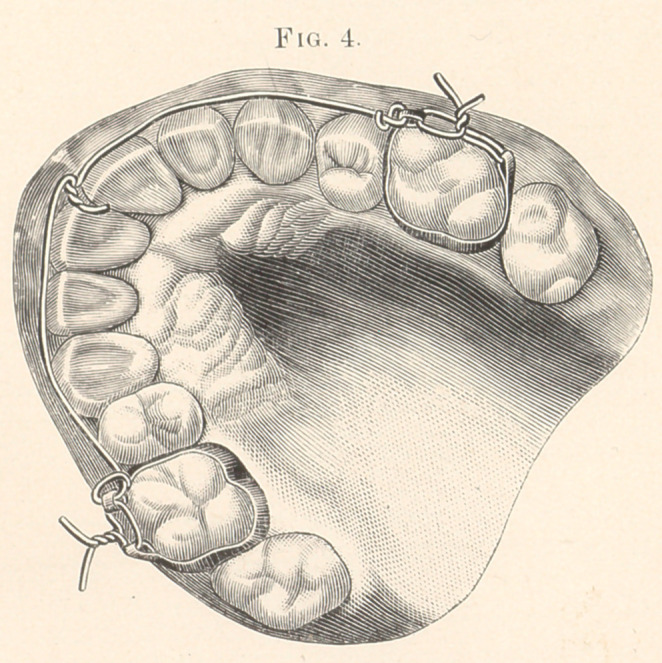


**Fig. 5. f5:**